# Time-Series Analysis and Healthcare Implications of COVID-19 Pandemic in Saudi Arabia

**DOI:** 10.3390/healthcare10101874

**Published:** 2022-09-26

**Authors:** Rafat Zrieq, Souad Kamel, Sahbi Boubaker, Fahad D. Algahtani, Mohamed Ali Alzain, Fares Alshammari, Fahad Saud Alshammari, Badr Khalaf Aldhmadi, Suleman Atique, Mohammad A. A. Al-Najjar, Sandro C. Villareal

**Affiliations:** 1 Department of Public Health, College of Public Health and Health Informatics, University of Ha’il, Ha’il 55476, Saudi Arabia; 2 Department of Computer & Network Engineering, College of Computer Science and Engineering, University of Jeddah, Jeddah 21959, Saudi Arabia; 3 Department of Health Informatics, College of Public Health and Health Informatics, University of Ha’il, Ha’il 55476, Saudi Arabia; 4 Department of Health Management, College of Public Health and Health Informatics, University of Ha’il, Ha’il 55476, Saudi Arabia; 5 Department of Public Health Science, Faculty of Landscape and Society, Norwegian University of Life Sciences,1430 Ås, Norway; 6 Department of Pharmaceutical Science and Pharmaceutics, Faculty of Pharmacy, Applied Science Provate University, Al Arab St 21, Amman 11118, Jordan; 7 Medical-Surgical and Pediatric Nursing Department, College of Nursing, University of Ha’il, Ha’il 55476, Saudi Arabia

**Keywords:** COVID-19, data analytics, Times series, ARIMA, Prophet, baseline, modeling, prediction

## Abstract

The first case of coronavirus disease 2019 (COVID-19) in Saudi Arabia was reported on 2 March 2020. Since then, it has progressed rapidly and the number of cases has grown exponentially, reaching 788,294 cases on 22 June 2022. Accurately analyzing and predicting the spread of new COVID-19 cases is critical to develop a framework for universal pandemic preparedness as well as mitigating the disease’s spread. To this end, the main aim of this paper is first to analyze the historical data of the disease gathered from 2 March 2020 to 20 June 2022 and second to use the collected data for forecasting the trajectory of COVID-19 in order to construct robust and accurate models. To the best of our knowledge, this study is the first that analyzes the outbreak of COVID-19 in Saudi Arabia for a long period (more than two years). To achieve this study aim, two techniques from the data analytics field, namely the auto-regressive integrated moving average (ARIMA) statistical technique and Prophet Facebook machine learning technique were investigated for predicting daily new infections, recoveries and deaths. Based on forecasting performance metrics, both models were found to be accurate and robust in forecasting the time series of COVID-19 in Saudi Arabia for the considered period (the coefficient of determination for example was in all cases more than 0.96) with a small superiority of the ARIMA model in terms of the forecasting ability and of Prophet in terms of simplicity and a few hyper-parameters. The findings of this study have yielded a realistic picture of the disease direction and provide useful insights for decision makers so as to be prepared for the future evolution of the pandemic. In addition, the results of this study have shown positive healthcare implications of the Saudi experience in fighting the disease and the relative efficiency of the taken measures.

## 1. Introduction

The COVID-19 pandemic is the most significant global crisis since the second world war. The disease is currently spreading across the globe at a surprisingly faster rate, affecting more than 213 countries, infecting more than 545,900,772 people and leading to 6,343,950 deaths worldwide as of 22 June 2022 according to the World Health Organization (WHO) [1].

**Figure 1:**
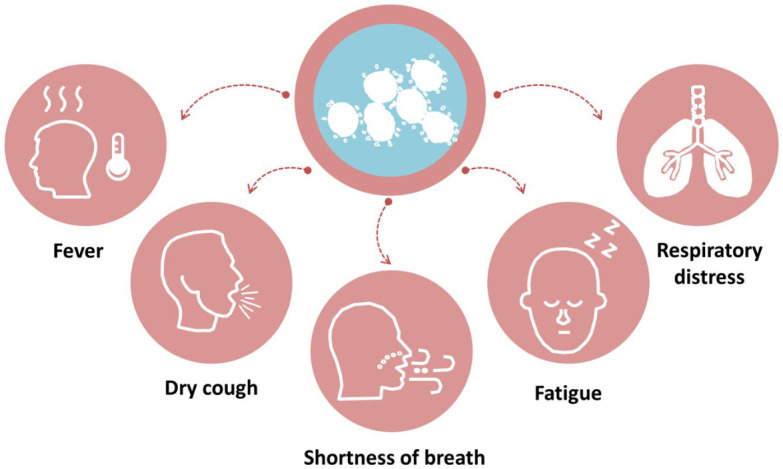
COVID-19 Symptoms.

COVID-19 is an infectious disease caused by the emergence of the new coronavirus in Wuhan, China, in December 2019. Four to five days after a person contracts the virus, symptoms typically appear. However, in some cases, the onset of symptoms can take up to two weeks. Some individuals never even exhibit any symptoms. The most common symptoms of COVID-19 are fever, cough, shortness of breath, fatigue, shaking chills, muscle pains, headaches, sore throats, runny or stuffy noses, and issues with taste or smell (Figure 1). If a patient has some of the symptoms presented in Figure 1, they are asked to test immediately. Saudi Arabia adopted the two tests approved by the American Food and Drug Administration (FDA) for diagnosing COVID-19, namely the Reverse Transcription Polymerase Chain Reaction (RT-PCR) and Antigen tests. RT-PCR is also called a molecular test. It detects the genetic material of the virus using a lab technique called reverse transcription polymerase chain reaction. A medical expert will take a fluid sample from the back of a patient’s nose by inserting a nasal swab into his nostril. When properly conducted by a medical specialist, RT-PCR tests are quite accurate; however, the quick test may miss some cases. If the patient is infected with a virus at the time of the test, results will reveal its presence. Even when the patient is no longer sick, the test may still be able to find remnants of the virus. The Antigen test detects certain proteins in the virus. This test is fast but it is less accurate than PCR. There is a higher likelihood of false-negative results, which means it is possible to have the viral infection but have a negative result. Depending on the circumstances, the medical professional might advise performing an RT-PCR test to confirm a negative antigen test result.

The disease symptoms encountered in Saudi Arabia were found to be common and similar to those encountered worldwide. As illustrated in Figure 1, such symptoms subside in a few days to weeks. However, a small percentage of infected persons develop severe illnesses and lose the ability to breathe on their own. In extreme circumstances, their organs fail, which can be fatal. The disease effect depends on the age and health of the infected individual. In fact, people who have cardiac illness, chronic obstructive pulmonary disease, asthma, high blood pressure, or weakened immune systems may experience severe problems. In some cases, the COVID-19 virus can cause death. In addition to the previous health consequences, the spread of the disease has also led to severe effects on social and economic systems [2,3,4]. In fact, due to stopping many economic activities, several persons have lost their jobs and many companies have closed. The disease’s spread dynamics can be explained by several factors including the demographic distribution of the population, the efficiency of the public healthcare system, the mitigation countermeasures taken by local health authorities and the availability of vaccines, among other factors. The COVID-19 pandemic’s evolution, like that of earlier pandemics, is not entirely random. The path of the disease looks like a life cycle, with the outbreak followed by an acceleration phase, an inflection point, a deceleration phase, and finally a stop or termination. The occurrence of novel variants of the virus such as Omicron or Delta, as well as other factors such as the organization of vaccination campaigns, may be all linked to the disease spread. Due to preventative measures, such as lockdowns and social distancing, the disease life cycle may differ from one country to another, and various countries may be in different phases at a given moment. In the same country, the disease might sometimes present several features depending on the region, most likely as a result of sociological and climatic factors. Research is currently being undertaken using various mathematical models to forecast the progression of the pandemic and to characterize its dynamics in order to aid in understanding its trajectory through time. The Susceptible-Infectious-Removed (SIR) class of compartmental modelling techniques, developed by Kermack and McKendrick [5] almost a century ago, is one of those popular models. SIR has played a key role in treating infectious diseases and continues to do so. The “Susceptible”, “Infectious”, and “Removed” percentages of a given population are divided into compartments. These compartments are related by dynamic interactions that are represented by non-linear ordinary differentials (ODEs). Authors in [6,7,8] used SIR models to forecast the COVID-19 outbreak, respectively, in India, Algeria and Saudi Arabia. The simulation results showed the necessity of interventions in flattening the disease propagation curve, delaying the peak, and lowering the fatality rate.

In order to predict the spread of COVID-19, it was discovered that the “econometric models” family of models was effective. The time series Auto-Regressive-Integrated-Moving-Average (ARIMA) model is the most well-known member of this family. The prevalence and incidence of COVID-19 were predicted by the authors in [9] using the ARIMA model and the Johns Hopkins epidemiological data. Authors in [10,11,12,13,14] performed forecasting of the spread of the COVID-19 pandemic using the ARIMA prediction model under current public health interventions, respectively, in Saudi Arabia, Kuwait, Egypt, Korea and Morocco. The prediction accuracy of the ARIMA model was found to be acceptable and adequate. Authors in [15] came to the conclusion that the ARIMA model, when compared to the AR (Auto Regression) model, provides the best match for predicting new cases in India. The authors of [16] sought to create the best models to anticipate new daily instances. The daily new cases in India and the US were fitted using ARIMA and a hybrid ARIMA model. In India, the ARIMA model’s predictive values were the most accurate; however, in the US, the hybrid ARIMA model performed better.

As an alternative to epidemiological and time series models, machine learning models showed potential in predicting COVID-19, as they did for modeling other outbreaks. References [17,18,19,20] included an overview of research using mathematical, machine learning and Deep Learning models to detect, diagnose, or forecast COVID-19. Authors in [21,22,23,24] used Support Vector Machine, variants of Recurrent neural network (RNN) and variants of long-short term memory (LSTM) for predicting COVID-19. It was found that these algorithms were effective due to the non-linear nature of how they handle the datasets.

References [25,26,27] trained the Facebook Prophet model in order to examine and predict the number of COVID-19 cases and fatalities based on the previously available data.

From the above literature review, a plethora of techniques from the field of statistics, data science, machine learning and artificial intelligence [28] have been used for COVID-19 prediction. To the best of the authors’ knowledge, the accuracy of the time-series and machine learning models are sensitive to the case study as well as to the time window covered by the utilized datasets. Saudi Arabia is a country of rapid economic growth, visited annually by millions of Muslim people for performing Hajj and Umrah and hosting immigrants from different countries, cultures and religions. Saudi Arabia also has a large surface territory exhibiting various climatic conditions. Therefore, Saudi Arabia includes several patterns making it a suitable case study that can represent Arabic, Muslim, developing and Oil-Producer countries. In addition, Saudi Arabia has had a relatively successful experience in mitigating the disease.

For the reasons cited above, this study’s main aim is to investigate statistical and machine learning-inspired time series approaches for modeling/analyzing the spread of COVID-19 in Saudi Arabia in terms of numbers of confirmed, recovered and death cases. More specifically, ARIMA and Facebook’s Prophet approaches were developed and then compared. In addition, healthcare implications regarding the Saudi experience in facing the disease will be analyzed according to the time-line evolution and more particularly regarding the events related to the countermeasures taken. Saudi Arabia announced on Monday 13 June 2022, that it is relaxing the restrictions on the use of face masks in enclosed spaces with the exception of the Grand Mosque in Mecca and the Prophet’s Mosque in Medina, as well as medical facilities, public gatherings, sporting events, flights, and public transportation. This study aims to evaluate the future short-term impacts of such a decision.

The rest of this paper is structured as follows. In Section 2, a data description and the proposed methods are introduced. Detailed experiments are outlined in Section 3.

## 2. Materials and Methods

### 2.1. Data Description

The datasets used in this study were collected from the official website of Saudi Ministry of Health [29] and from the dashboard (/www.https://covid19.moh.gov.sa/, accessed on 1 August 2022). It contains the daily number of confirmed, recovered and death cases from 2 March 2020 to 22 June 2022.

**Figure 2:**
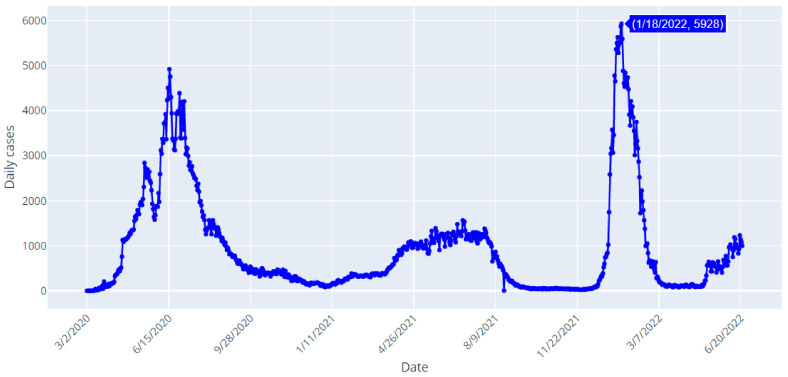
COVID-19 Confirmed Cases in Saudi Arabia from 2 March 2020 to 22 June 2022.

**Figure 3:**
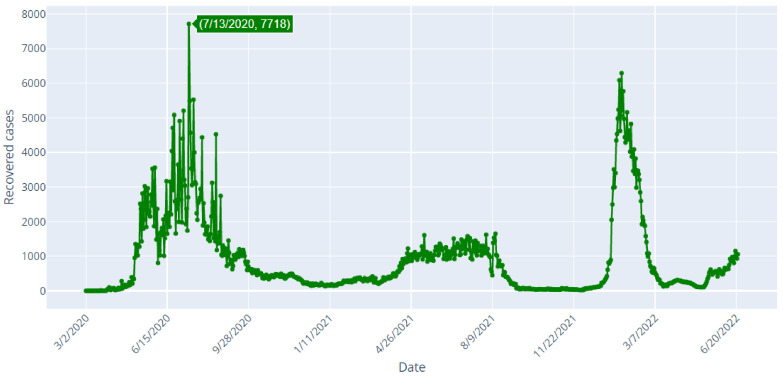
COVID-19 Recovered Cases in Saudi Arabia from 2 March 2020 to 22 June 2022.

**Figure 4:**
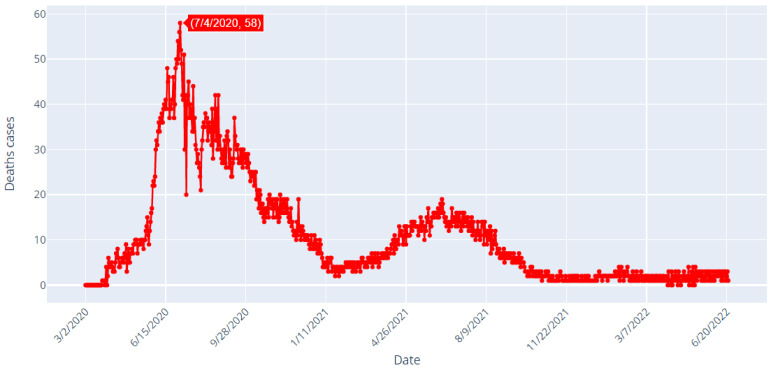
COVID-19 Deaths in Saudi Arabia from 2 March 2020 to 22 June 2022.

Table 1 shows data samples and Figure 2, Figure 3 and Figure 4 depict the daily confirmed (blue), recoveries (green) and deaths (red) cases of COVID-19 in Saudi Arabia from 2 March 2020 to 22 June 2022.

Figure 2 shows that the first confirmed case was reported on 2 March 2020. The number of confirmed cases reached 1002 cases on 22 June 2022. The highest daily number of confirmed cases is 5928 cases, and it was reported on 18 January 2022.

Figure 3 shows that the highest daily number of recovered cases is 7718 cases, and it was reported on July 13, 2020. The number of recovered cases reached 1059 cases on 22 June 2022.

Figure 4 shows that the highest daily number of deaths is 58, and it was reported on 4 July 2020. The number of deaths reached 1 case on 22 June 2022. If we analyze the spread of COVID-19 in Saudi Arabia, as it is shown in Figure 2, Figure 3 and Figure 4, we can clearly detect that there was a fluctuating behavior which may reveal dynamics that are difficult to model. Overall, there have been 789,296 confirmed cases, 770,077 recovered and 9195 deaths in Saudi Arabia, up to the date of writing this manuscript (23 June 2022). In Table 2, the major events that marked the pandemic in Saudi Arabia are shown. The majority of these events were highly related to the mitigating actions taken by the Saudi authorities. In addition, the evolution of the numbers of new infections and deaths occurring after those events may show that the measures taken exhibited positive effects on the disease spread. Moreover, other actions’ effects appeared after a few days or even weeks.

### 2.2. Models

COVID-19 time series (TS) data, like other TS, is simply a collection of data recorded over a period of time usually regularly spaced (daily, weekly, monthly, *…*). TS are often analyzed to understand the past, in order to predict the future (forecast). TS are mainly employed for helping managers and policy makers to make well-informed and sound decisions. TS can be univariate when its values are taken by a single variable at a periodic time instance over a period, and multivariate when its values represent multiple variables at the same periodic time instances over a period. TS data are different to cross-sectional data which record individuals, companies or others at a single point in time.

A natural temporal ordering exists in TS data. The observations made in the past, often known as lag times or lags, are frequently of interest to data scientists. Observations that are close in time tend to be correlated, which is a characteristic of most time series that sets them apart from cross-sectional data. The aim is often to estimate how TS will evolve in the future, with time serving as the independent variable. In general, TS data can be found in any area of applied science and engineering that uses temporal observations, including social sciences, finance, economics, epidemiology, and more. There are a few factors such as trend, stationarity, seasonality and correlation that are relevant when dealing with time series. When there is a long-term rise or fall in the data, the situation is referred to as a trend. Stationarity is another crucial property of time series. If a time series’ statistical characteristics remain constant across time, it is said to be stationary. Its mean and variance are constant, and its covariance is time-independent [30]. Seasonality is the existence of recurring fluctuations that occur at predetermined regular periods of less than a year. The term “autocorrelation” describes the similarity of data as a function of their distance in time.

A time series analysis is the process of examining time series data to extract useful statistics and other aspects of the data, and time series forecasting is the process of using a model to project future values based on observed values. The “No-Free-Lunch Theorem” [31] states that no forecasting technique is optimal for every time series. Instead, the data analysis expert must choose a forecasting methodology from one of the three families of forecasting techniques listed below: (1) machine learning, (2) statistical models, and (3) hybrid methods [32].

In this paper, for forecasting the COVID-19 time series, we developed and compared three approaches based on ARIMA, Facebook’s Prophet and baseline models known to be relatively simple and possessing good performance. Details of these models are provided in the following sections.

#### 2.2.1. ARIMA Model

The ARIMA model is the acronym of the Autoregressive Integrated Moving Average model. It is also known as the Box–Jenkins model. The ARIMA model is the most widely used approach to univariate time series forecasting. It is composed of three key components [33].

- AR (Autoregression): This component of ARIMA expresses the dependent relationship between the current observation and a number of lagged observations.
  (1) $$y_{t}=C+\alpha_{1}y_{t-1}+\alpha_{2}y_{t-2}+\cdot \cdot \cdot +\alpha_{p}y_{t-p}$$
  where *C* is a constant; $y_{t-1},y_{t-2},y_{t-p}$ are the lags (past values); and $\alpha_{1},\alpha_{2},\alpha_{p}$ are lag coefficients which are estimated by the model.
- I (Integrated): This term refers to the use of a differencing operator of raw observations (e.g., subtracting an observation from an observation at the previous time step) in order to make the time series stationary.
- MA (Moving Average): This part of the ARIMA model describes the dependency between the current observation and a residual error from a moving average model applied to lagged observations.
  (2) $$y_{t}=\epsilon_{t}+\beta_{1}\epsilon_{t-1}+\beta_{2}\epsilon_{t-2}+\cdot \cdot \cdot +\beta_{q}\epsilon_{t-q}$$
  where $\epsilon_{t},\epsilon_{t-1},\epsilon_{t-q}$ are white noise terms for the respective lags, i.e,. $y_{t-1},y_{t-2},y_{t-q}$; $\beta_{1},\beta_{2},\beta_{q}$ are the parameters of the model.

The ARIMA model is characterized by the order of each of these components. Following the notation ARIMA (*p*, *d*, *q*), the model parameters are described as follows [34]:

- *p* is the number of autoregressive lags included in the model;
- *d* is the order of differencing used to make the data stationary;
- *q* is the number of moving average lags included in the model.

**Figure 5:**
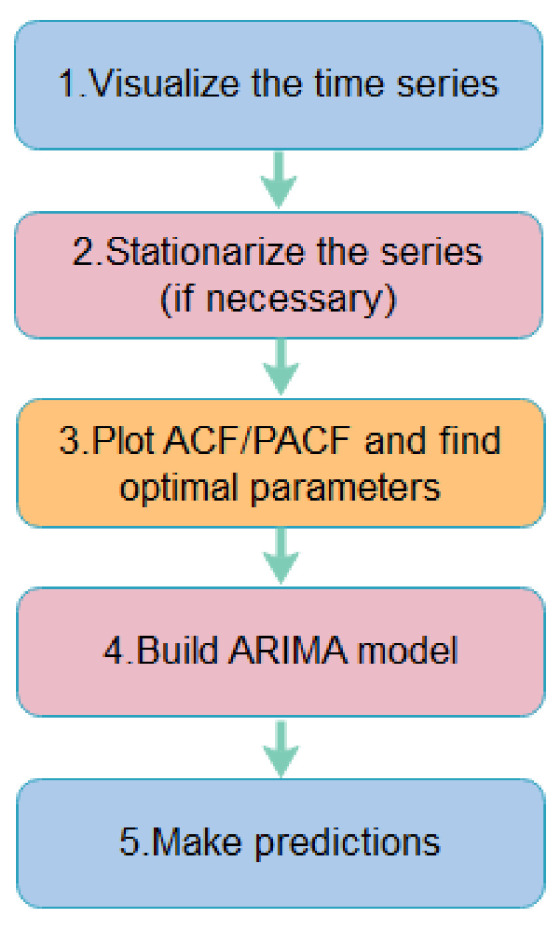
The general procedure to fit an ARIMA model.

There are many heuristics for choosing the parameters of an ARIMA model. One popular method is the Box–Jenkins method, which is an iterative multistep process (Figure 5). In order to determine p and q, the autocorrelation function (ACF) and partial autocorrelation function (PACF) provide guidance for the autoregressive and moving average orders that are appropriate for the considered model. In this paper, a grid search of hyperparameters is used to tune the ARIMA model.

For further details about ARIMA models and time series, the interested reader can refer to the following books: [35,36,37].

#### 2.2.2. Facebook’ Prophet

On February 23, 2017, Prophet, a method for predicting time series data, was published by Facebook and made available for use. Prophet is a robust forecasting technique. It can be easily used by users without a strong background in time series forecasting. This tool helps produce accurate forecasts for a wide range of problems. Based on an additive model, Facebook’s Prophet fits non-linear patterns with weekly and yearly seasonality as well as considering holidays patterns. The Prophet model includes in general three key elements [38]:

(3) $$y\left(t\right)=n\left(t\right)+p\left(t\right)+h\left(t\right)+\varepsilon_{t}$$

where:

- *n*(*t*): is the trend function which models non-periodic changes in the value of the time series;
- *p*(*t*): represents periodic changes (seasonality);
- *h*(*t*): represents the effects of holidays
- $\varepsilon_{t}$: an error term.

Two potential trend models are implemented by the Prophet library for *n*(*t*). The first type is referred to as nonlinear, saturating growth. It takes the shape of a logistic growth model.

(4) $$n\left(t\right)=\frac{C}{1+e^{-k(t-m)}}$$

where,

- *C*: carry capacity;
- *k*: growth rate;
- *m*: offset parameter.

The latter, however, is a straightforward Piece-wise Linear Model with a stable rate of growth.

(5) $$n=\left\{\begin{array}{cc} \beta_{0}+\beta_{1}x & x\leq c\\ \; & \\ \beta_{0}-\beta_{2}c+\left(\beta_{1}+\beta_{2}\right)x) & x>c \end{array}\right.$$

where,

- *c*: trend change point;
- β: trend parameter (can be tuned as per requirement).

For situations without excessive growth, the latter is the ideal option. Due to weekly and yearly seasonality, the seasonal component *p*(*t*) offers a flexible model of periodic variations. Fourier series are used in Prophet’s yearly seasonality model.

(6) $$p\left(t\right)=\sum_{n=1}^{N}\left(a_{n}cos\left(\frac{2\Pi nt}{P}\right)+b_{n}sin\left(\frac{2\Pi nt}{P}\right)\right)$$

where,

- *P*: regular period expected for considered time series;
- It was discovered that *N* = 10 and *N* = 3, respectively, for yearly and weekly seasonality, work effectively for the majority of cases. A model selection method such as AIC could be used to automate the selection of these parameters.

Black Fridays and other predictable exceptional days with irregular schedules are represented by the component *h*(*t*). The data analyst must supply a customized set of events in order to make use of this feature. The information that was not taken into account by the model is represented by the error term which reflects the model robustness $\varepsilon_{t}$. A uniformly distributed noise is typically used to model it [38].

**Figure 6:**
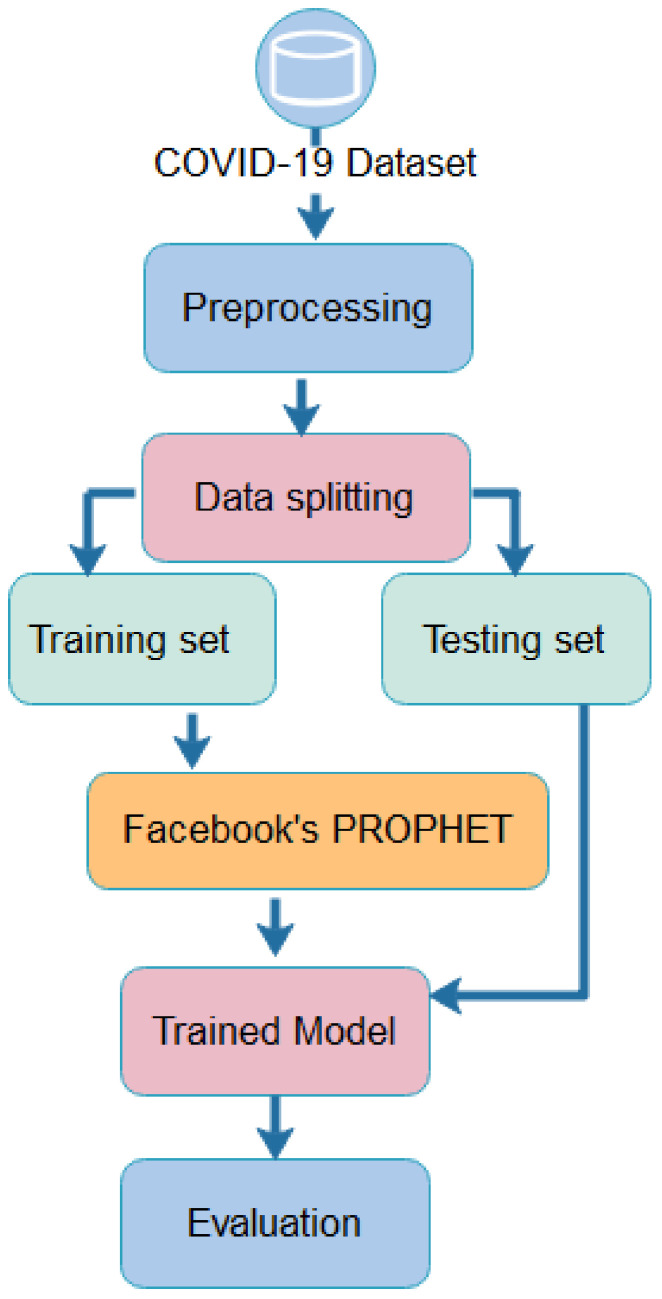
Forecasting using Facebook’s Prophet.

Prophet, a novel time series forecasting model from the machine learning family, adheres to the streamlined framework shown in Figure 6.

### 2.3. Metrics for Evaluation

The following three criteria (Mean Absolute Error (MAE), Root Mean Squared Error (RMSE) and the coefficient of determination (R^2^)) were applied to each case (confirmed, recovered and deaths) to compare the goodness-of-fit yielded by the investigated models:

(7) $$MAE=\frac{1}{n}\sum_{t=1}^{n}\left|\hat{y_{t}}-y_{t}\right|$$

(8) $$RMSE=\sqrt{\frac{1}{n}\sum_{t=1}^{n}{\left(\hat{y_{t}}-y_{t}\right)}^{2}}$$

(9) $$R^{2}=1-\frac{\sum_{t=1}^{n}{\left(\hat{y_{t}}-y_{t}\right)}^{2}}{\sum_{t=1}^{n}{\left(\bar{y}-y_{t}\right)}^{2}}$$

where $y_{t}$ is the actual value, $\hat{y_{t}}$ is the predicted value and $\bar{y}=\frac{1}{n}\sum_{t=1}^{n}y_{t}$ is the mean of $y_{t}$. These three measures can be used to assess a model’s performance and depict its accuracy very well. These three metrics can be computed for each model and compared to each other to identify the most accurate one. The fit is better when MAPE and MSE have smaller values and the coefficient of determination has a value close to 1 which represents the ideal fit.

## 3. Results

Table 3 lists the hardware and software specifications involved in the experiments conducted in this work. Table 3 shows the packages used during the implementation of ARIMA, Prophet and baseline models.

### 3.1. Results of ARIMA Models

Checking the stationarity data is the initial step in time series forecasting because the majority of TS models rely on this assumption. Furthermore, compared to non-stationary data, the stationary TS theory is better established and simpler to put into practice. In time series analysis and forecasting, visualization is crucial. Line plots of datasets, for instance, can help to detect patterns, cycles, and seasonality. As a result, this can affect the model choice. The stationarity can be more easily seen on a line plot. If a time series’ statistical characteristics do not alter over time, it is classified as stationary. Its mean and variance are therefore constant, and its covariance is not affected by time [30]. To examine stationarity in TS data, a variety of statistical methods are available. The Augmented Dickey–Fuller (ADF) test, also referred to as a unit root test, is one of the most popular types [33]. In this test, we assume that TS is not stationary, which is the null hypothesis. A Test Statistic and a few crucial values for various confidence levels are included in the test results. We can reject the null hypothesis and declare that the series is stationary if the test statistic is less than the critical value. Equivalently, the null hypothesis can be rejected if the p-value is less than 0.05. For statistical modeling techniques to succeed, the TS must be stationary. If a TS is non-stationary, differencing (the d parameter of ARIMA (*p*,*d*,*q*)) provides a straightforward technique to make it stationary. Finding *p* and *q* for ARIMA is the next task after TS becomes stationary. The ARIMA (*p*,*d*,*q*) model can be expressed mathematically as follow [39]:

(10) $$\Phi {\left(L\right)}^{p}\Delta^{d}y_{t}=\varphi {\left(L\right)}^{q}\Delta^{d}\epsilon_{t}$$

(11) $$\Delta^{d}y_{t}=y_{t}^{d-1}-y_{t-1}^{d-1}$$

where $y_{t}$ is the time series; *p*, *d*, and *q* are, respectively, the order of AR, order of integration (number of differences) and MA components of the ARIMA model. $\Delta^{d}$ is an operator to make $y_{t}$ stationary; *L* is defined as the lag operator; $\Phi {\left(L\right)}^{p}$ is the lag polynomials of order *p*, *q* is the number of time lags of the error term to regress on, φ is defined analogously to Φ and $\epsilon_{t}$ is a white noise.

Through Auto Correlation (ACF) and Partial Auto Correlation (PACF) graphs, we can learn some important properties of the TS data since the ACF measures the linear relationships between observations at different lags and PACF measures the partial correlation between two points at a specific lag of time. Alternatively, a widely used method for estimating the three parameters required in ARIMA(p,d,q) is the grid search procedure. In order to discover how to tune the ARIMA model using grid search of hyperparameters, the reader can refer to [33]. Once p, d and q are carefully chosen and fixed, one has to fit the data to the ARIMA which has to be finalized so as to make predictions on new data. The skill and the capability of the forecast model can be evaluated through performance measures (See Section 2.3).

#### 3.1.1. Prediction of Confirmed Cases

**Figure 7:**
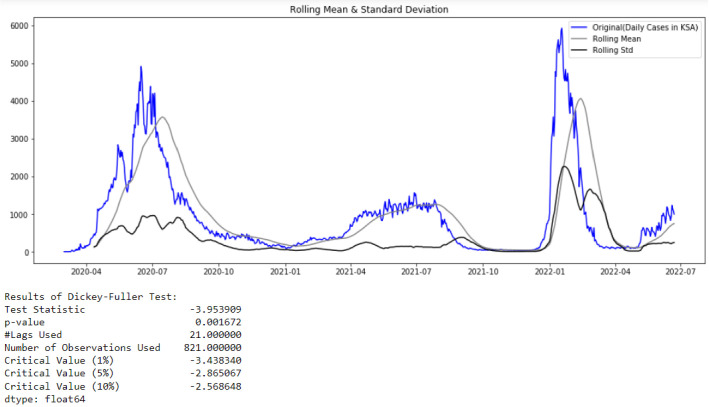
Test of Stationarity of Confirmed Cases data.

When referencing Figure 2 of the confirmed cases in Saudi Arabia from 2 March 2020 to 22 June 2022, it can be observed that the TS is stationary. This observation can be proved by the results of the Augmented Dickey–Fuller test shown in Figure 7. Here, in order to optionally specify the number of lags considered in the ADF test, the Akaike’s Information Criterion ‘AIC’ is used through the optional parameter autolag = ‘AIC’ ( in the python function *adfuller*()). Usually, to identify the best balance between variance and bias, the complexity of the models is penalized using the AIC information criteria.

In Figure 7, the rolling mean of a time series for time point t for a window size w is simply the mean of the previous w time steps and the rolling standard deviation for the same example is defined as the standard deviation of the previous w time. If the rolling statistics do not fluctuate appreciably over time, a time series is said to be “visually stationary”.

**Figure 8:**
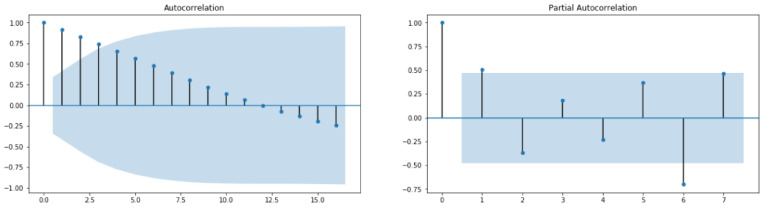
ACF and PACF for Cases data.

From Figure 8, it can also be concluded that the studied time series comprising confirmed cases in Saudi Arabia from 2 March 2020 to 22 June 2022 is stationary (interpret the test using the p-value or the critical values returned by the test: test statistic is lower than the critical value).

**Figure 9:**
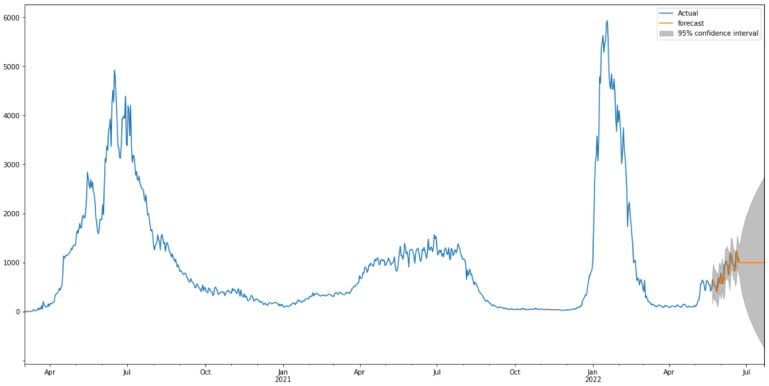
Predicted vs. Expected values for confirmed cases.

Figure 9 shows the predictions of COVID-19 case trends with the ARIMA model. As is shown in Figure 9, the model fits the confirmed cases in Saudi Arabia very well, with the values and the curve itself being very close to the actual ones. Table 4 shows the three metrics of the ARIMA model. MAE and $R^{2}$ of ARIMA used for confirmed cases are greatly improved compared to the baseline model with relatively low values. However, the RMSE of the ARIMA model has slightly worse performance than the baseline model.

#### 3.1.2. Prediction of Recovered Cases

**Figure 10:**
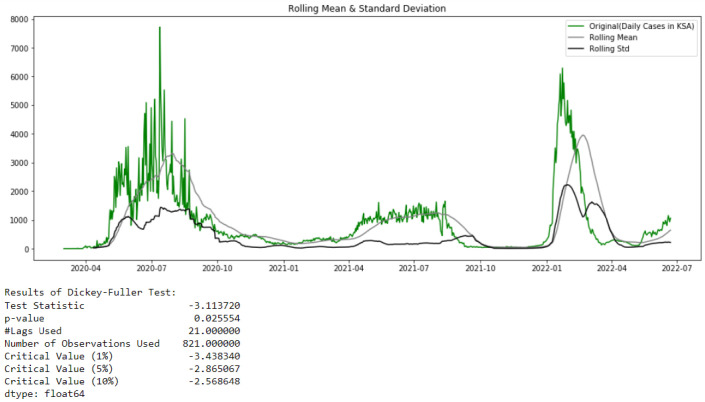
Test of Stationarity of Recovered Cases data.

When observing Figure 3 of recovered cases in Saudi Arabia from 2 March 2020 to 22 June 2022, it can be noted that the studied TS is stationary. This observation can be proved by the results of the Augmented Dickey-Fuller test shown in Figure 10.

**Figure 11:**
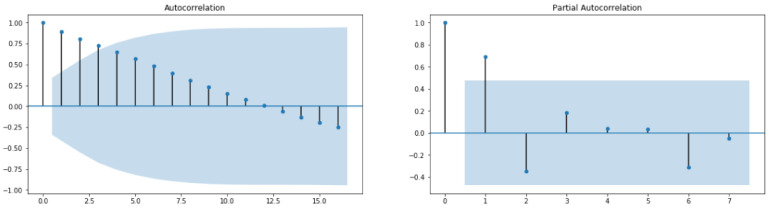
ACF and PACF for Recovered Cases data.

From Figure 10, it can be concluded that the studied time series comprising recovered cases in Saudi Arabia from 2 March 2020 to 22 June 2022 is stationary (test statistic is lower than critical value; therefore, the considered data require some transformations). By looking at the autocorrelation function (ACF) and partial autocorrelation (PACF) plots (Figure 11), the numbers of AR and/or MA terms that are needed can be tentatively identified.

**Figure 12:**
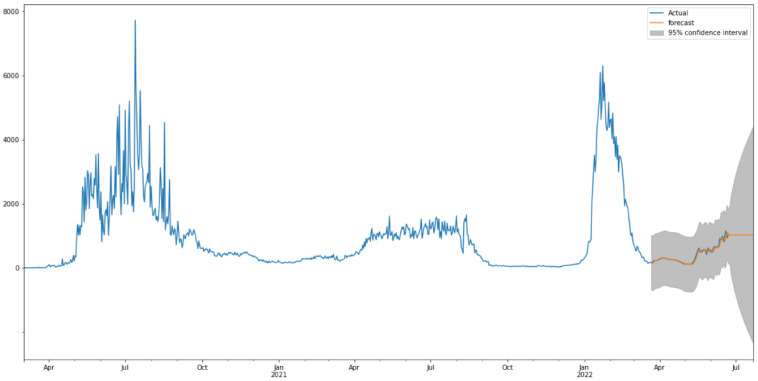
Predicted vs Recorded values for recovered cases.

Figure 12 shows the predictions of COVID-19-recovered cases with the ARIMA model. As is shown in Figure 12, the model fits the recovered cases in Saudi Arabia very well, with the values and the curve itself being very close to the actual ones. Table 5 shows the three metrics of the ARIMA model. $R^{2}$ of ARIMA used for recovered cases is greatly improved compared to the baseline model with relatively low values. However, the RMSE and MAE of the ARIMA model has slightly worse performance than the baseline model.

#### 3.1.3. Prediction of Deaths

**Figure 13:**
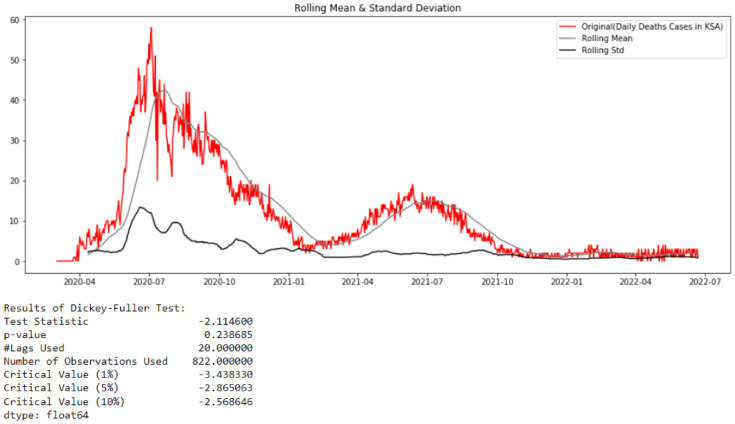
Test of Stationarity of Deaths Cases data.

When observing Figure 4, representing the deaths cases in Saudi Arabia from 2 March 2020 to 22 June 2022, it can be noted that the studied TS is non-stationary. This observation can be proved by the results of the Augmented Dickey–Fuller test shown in Figure 13.

**Figure 14:**
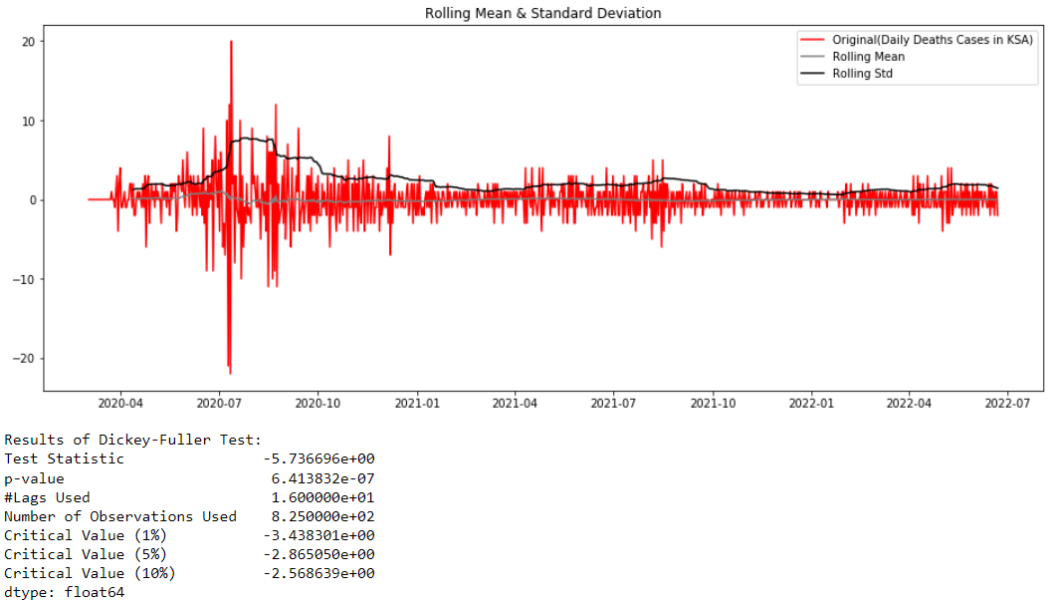
Test of Stationarity of Deaths Cases data after differencing.

From Figure 13, it can be concluded that the studied time series comprising deaths cases in Saudi Arabia from 2 March 2020 to 22 June 2022 is non-stationary (test statistic is greater than critical value; therefore, the TS data need to be stationary). By looking at the autocorrelation function (ACF) and partial autocorrelation (PACF) plots of the differenced series (Figure 14), the numbers of AR and/or MA terms that are needed can be tentatively identified.

**Figure 15:**
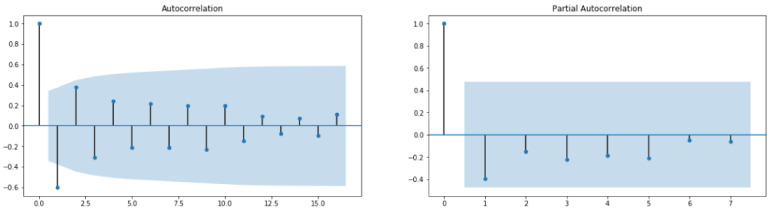
ACF and PACF for Deaths Cases data.

**Figure 16:**
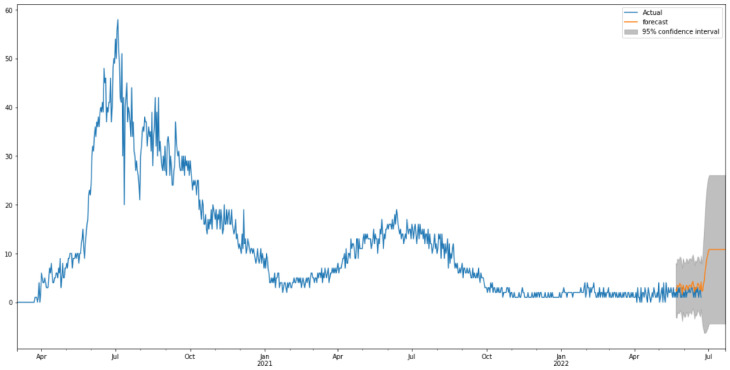
Predicted vs. Actual values for death cases.

By looking at the autocorrelation function (ACF) and partial autocorrelation (PACF) plots of the differenced series (Figure 15), we can tentatively identify the numbers of AR and/or MA terms that are needed. In this case, an initial order for the model will be (1, 0, 1); however, after performing a grid search for hyperparameters of the ARIMA model, we found that the best fitted order is (0, 0, 9). Using the latter model provided the forecast illustrated in Figure 16.

Figure 16 shows the predictions of COVID-19 death cases with the ARIMA model. As is shown in Figure 16, the model fits the death cases in Saudi Arabia very well, with the values and the curve itself being very close to the actual ones. Table 6 shows the three metrics of the ARIMA model. $R^{2}$ of ARIMA used for death cases is greatly improved compared to the baseline model with relatively low values. However, the RMSE and MAE of the ARIMA model have slightly worse performance than the baseline model.

### 3.2. Results of Prophet

When using Prophet under a Python environment, an instance of the *Prophet* class should be created and then its *fit* and *predict* methods are recalled. The input to Prophet is always a data frame with the following two columns: **ds** and **y**. The **ds** (datestamp) column should be of a format of a date or a timestamp expected by Pandas while **y** column must be numeric, and represents the variable expected to be forecasted.

#### 3.2.1. Prediction of Confirmed Cases

**Figure 17:**
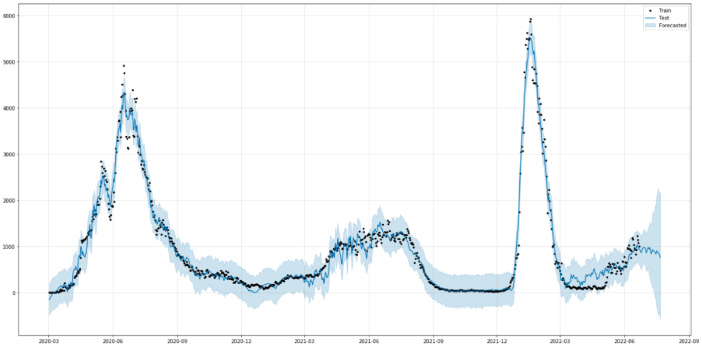
Predicted and actual values of Confirmed Cases.

**Figure A1:**
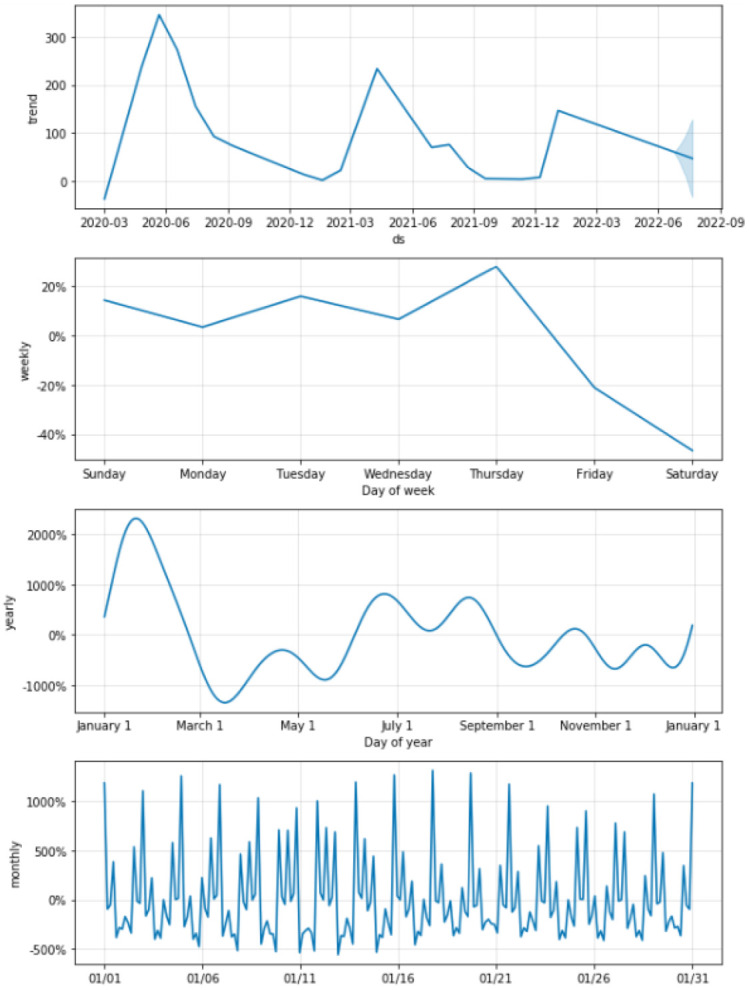
Components of Confirmed Cases.

Figure 17 shows the relationship between the original values (black dots) of confirmed cases in Saudi Arabia from 2 March 2020 to 22 June 2022 and the predicted values (blue solid line). The predicted and the actual values are close to one another as seen in Figure 17. We can also see the forecast components (Figure A1) by using the Prophet.plot-components method. This method provided the analysis of the trend of COVID-19 confirmed cases in Saudi Arabia until 22 June 2022, on a weekly and monthly basis. Table 7 shows the three performance metrics of the Prophet model. $R^{2}$ of the Prophet used for confirmed cases is clearly better than the baseline model (0.977 against 0.734 in a scale ranging from 0 to 1 which corresponds to the ideal fit). In terms of RMSE and MAE, the Prophet model provided a slightly worse performance than the baseline model.

#### 3.2.2. Prediction of Recovered Cases

**Figure 18:**
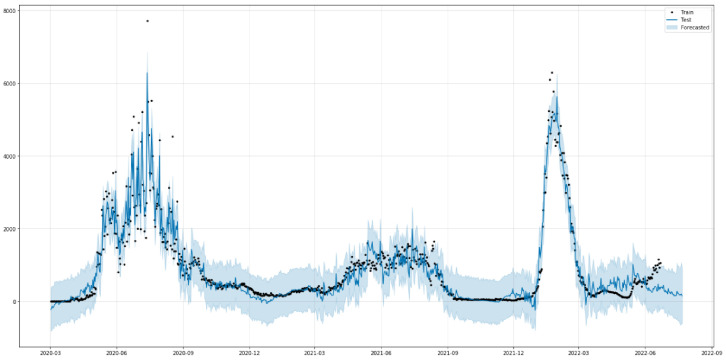
The predicted and original values of Recovered Cases.

**Figure A2:**
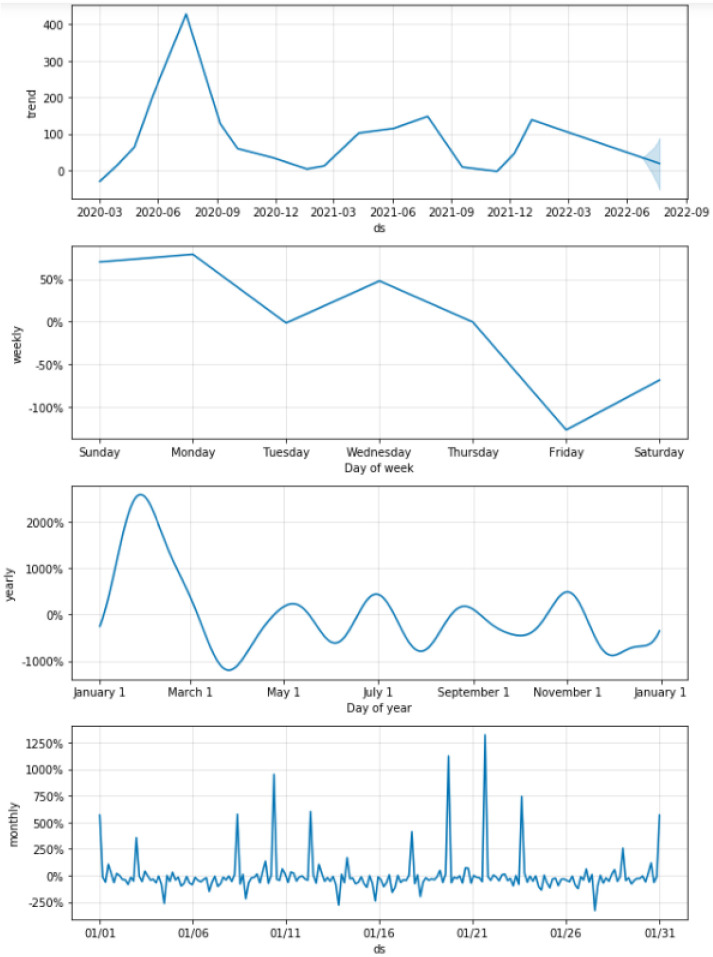
Components of Recovered Cases.

Figure 18 indicates the relationship between the original values (black dots) of recovered cases in Saudi Arabia from 2 March 2020 to 22 June 2022 and the predicted values (blue solid line). The predicted and the original values are similar, as seen in Figure 18. The trend of recovered cases, in a weekly, and monthly analysis of COVID-19 in Saudi Arabia until to 22 June 2022 is shown in Figure A2. Table 8 shows the three metrics of the ARIMA model. $R^{2}$ of ARIMA used for the recovered cases is greatly improved compared to the baseline model with relatively lower values. However, the RMSE and MAE of the ARIMA model have worse performance than the baseline model.

#### 3.2.3. Prediction of Deaths

**Figure 19:**
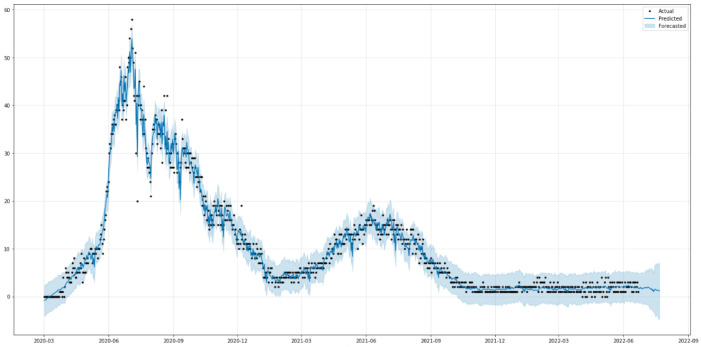
The predicted and actual values of Death Cases.

**Figure A3:**
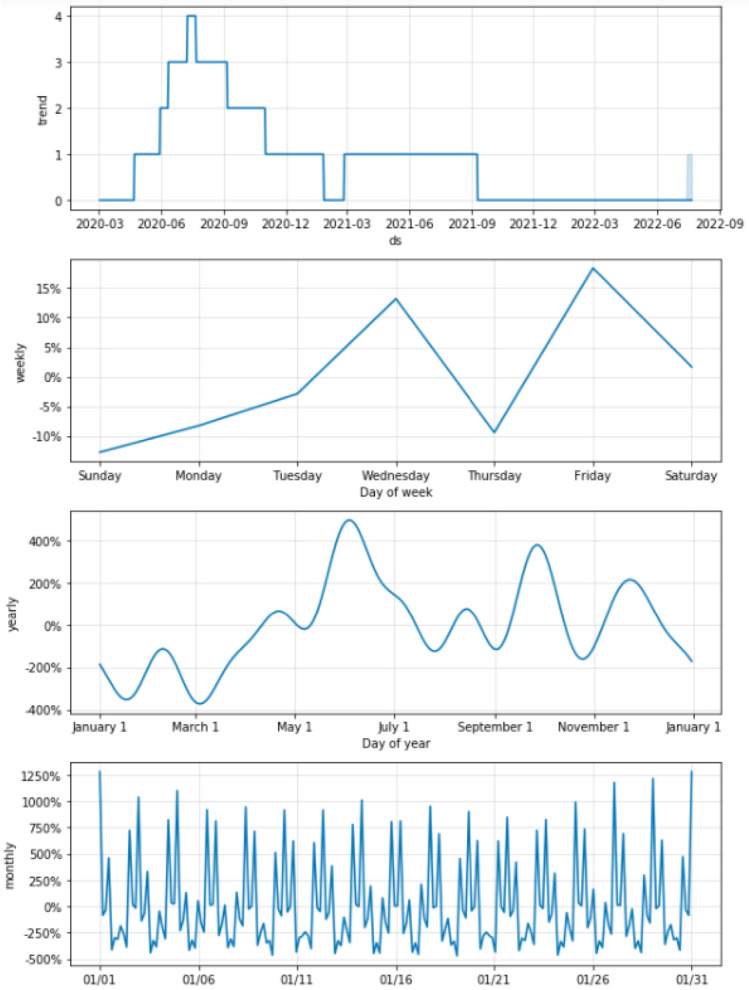
Components of Deaths Cases.

Figure 19 shows the relationship between the actual (black dots) deaths in Saudi Arabia from 2 March 2020 to 22 June 2022 and the predicted values (blue solid line). The predicted and the original values are very similar, as seen in Figure 19. The trend of death cases, on a weekly and monthly basis, is shown in Figure A3. Table 9 shows the three metrics of the ARIMA model. MAE and $R^{2}$ of the Prophet model used for death cases are obviously improved compared to those of the baseline model. The RMSE of the Prophet model has slightly worse performance than the baseline model.

## 4. Discussion

To date, over 615 million confirmed cases have been reported, and over 6 million people died worldwide. The physical and mental health of people is seriously impacted by COVID-19, which also has an impact on everyone’s lifestyle and the world economy. Given that COVID-19 is currently a serious global crisis, it is essential to fully comprehend the pandemic curve and forecast its future trajectory. An accurate COVID-19 forecast may have various advantages. It can assist in preserving lives, minimizing losses of financial resources, managing medical and human resources in healthcare, and reviving the world economy. The total number of cases of COVID-19 (or any other disease) is a common example of time series data, and there are currently a variety of analysis techniques to help identify patterns or forecast trends in such data. Time series data analysis has been proven to be successful with statistical methods (AR, MA, ARIMA, etc.), machine and deep learning methods (ANN, LSTM, etc.), and many other methods. Some of these techniques have already provided significant insights into the dynamics of infectious disease transmission and its surveillance. Records of new infections, recoveries and deaths are often released daily, which makes the task of predicting the future trend of the disease having lower performance when using data-demanding techniques such as deep learning (DL). However, statistical techniques such as ARIMA and straightforward machine learning (ML) techniques such as Facebook’s Prophet continue to show good performance. To the best of the authors’ knowledge, using ARIMA and Facebook’s Prophet with more than two years of COVID-19 pandemic data in Saudi Arabia represents the first study of its category. This relatively long period was marked by several events such as the vaccination campaigns organized over many countries, the emergence of new variants of the virus as well as the relaxation of lockdown measures and mask wearing. For every time series forecasting task, establishing a baseline is crucial. Usually, before starting any forecasting exercise, a baseline model should be first investigated. In that sense, the baseline model offers a basis for comparison for any additional forecasting approach. The newly developed approach should be modified or replaced if its performance is at the same level or below the baseline model. The persistence or the “Zero Rule” algorithm, also called the naive model, is the most often used. It uses the value at the current time step (t) to predict the expected outcome at the next time step (t + 1) [33]. In this study, Facebook’s Prophet and the ARIMA models are compared to the naive model. Table 10 includes the RMSE, MAE, and $R^{2}$ metrics for each model used in this study. The table clearly shows that the ARIMA model outperforms both Facebook’s Prophet and the baseline models at predicting confirmed and recovered cases of COVID-19 since it has the highest $R^{2}$ and the lowest MAE. However, the Prophet model performs better at predicting death cases. The ARIMA and Prophet model performances are comparable with almost similar results. Both models predict values that are practically equal to the actual values, which means they may be very useful in anticipating the number of confirmed, recovered and death cases in the future and provide individuals helpful recommendations on how to improve the COVID-19 mitigating measures. The optimal performance that ARIMA is capable of relies on a particular tuning parameter range. With a wide parameter selection, the model may identify better parameters for each of the cases under consideration; however, the amount of time and computational cost required to implement it may significantly increase. Under these conditions, data scientist may sacrifice an amount of the accuracy for quicker and simpler implementation of the model by using a restricted range of parameter selection. A reduced range of parameters for faster grid search (or similar approaches) can thus be used to speed up computing in some circumstances where the model accuracy does not need to be exceptionally high. Regarding the Prophet model, it has been found to present clear benefits and drawbacks. In fact, it requires the least amount of design and computing effort among ARIMA (for confirmed, recovered, and death cases). Furthermore, in this research, it has been found to be robust to missing data in a time series.

Although a fair comparison should be conducted on the same data set, covering the same period and the same location, results obtained in this study have been compared to those of three previous works in Saudi Arabia. The work in reference [10] used the ARIMA model but covered the beginning of the pandemic (From 2 March 2020 to 20 April 2020). The disease spread has after that shown many fluctuations. Our work is more beneficial to the work in [10] since it covered a period of more than two years where many aspects and virus variants appeared and highly impacted the disease dynamics. Reference [40] developed several growth models for the case study of Saudi Arabia. It obtained a coefficient of determination comparable to the performances of our present study. However, those models failed in predicting the probable end date of the disease. The work in [41] provided performances worst than those obtained in this study although it used sophisticated deep learning techniques shown to be data demanding.

In this study, the Prophet and ARIMA models produced reliable findings. However, given the complexity of the COVID-19 situation which exhibited concerns with virus mutation, population density, international travel, human behaviors, etc., the Prophet and ARIMA models are not well suited to handle such data trends. They occasionally perform worse than the baseline model in terms of RMSE and MAE (see Table 10). To overcome this issue, either multivariate time series forecasting or hybrid techniques may be implemented. Multivariate forecasting can be used to improve the thoroughness of the experiment and attain the best results since more data sources can improve accuracy. Recently, hybrid models ([42,43,44]) were used in time series analysis. For improved outcomes, these models typically incorporate machine learning techniques such as ANN (Artificial Neural Network) and statistical models including ARIMA. Future research can examine these models to improve their ability to forecast COVID-19.

## 5. Conclusions

When compared to other countries, the patterns from the most recent statistics demonstrated that the Saudi Arabian authorities’ quick and effective actions to restrict the pandemic imparted a beneficial effect, although numerous parameters affected the pandemic spreading in the country. This relatively successful experience in mitigating the disease provided this research with insights to deeply analyze and predict the time series data collected from official authorities. The evolution of COVID-19 using the Facebook’s Prophet and ARIMA models was investigated. The forecast was based on the data from 2 March 2020 until 22 June 2022. The results from these two models are not quantitatively different, since both models predicted a significant decrease in recovered cases and deaths in Saudi Arabia for the next month. For both models, the confirmed cases expected next month are in flux. These findings would aid the Saudi authorities in better containing the COVID-19 outbreak in the future. The results indicate that, although a one-size-fits-all approach does not exist, the Prophet model prevails since it is the easiest model to use and requires almost no manual effort. However, in the case of COVID-19, there are multiple issues involved such as virus mutation and human behaviors and such data cannot fit well in the Prophet model. These influential factors that may affect the disease dynamics need to be further investigated in a multi-variate time series context. Another improvement that should be investigated is the use of hybrid models which aggregate the benefits of different models, more specifically statistical and machine learning approaches.

## Figures and Tables

**Table 1:** COVID-19 Data in Saudi Arabia: Confirmed, Recovered, and Deaths.

Date	Confirmed	Recovered	Deaths
3 February 2020	1	0	0
...	...	...	...
4 July 2020	1247	1429	58
...	...	...	...
13 July 2020	2692	7718	40
...	...	...	...
...	...	...	...
18 January 2022	5928	4981	2
...	...	...	...
22 June 2022	1002	1059	1

**Table 2:** Major events in Saudi Arabia during the COVID19 Pandemic.

Event	Date
First case of COVID19	2 March 2020
Umrah suspension	3 March 2020
Test for COVID19 available for anyone with symptoms	5 March 2020
School closures	9 March 2020
Mosque closures	15 March 2020
Flights suspended to number of countries	9 March 2020
Gov/private suspension	14 March 2020
Domestic flights suspension	20 March 2020
Riyadh, Makkah and Madinah lockdown-curfew (6 am–3 pm)	23 March 2020
Jeddah areas lockdown-24 h curfew	24 March 2020
Makkah lockdown	25 March 2020
Emergence of the omicron variant	17 June 2020
COVID-19 Vaccines	15 December 2020
Reopening of schools and universities	28 August 2021
Saudi Arabia ended its COVID-19 restrictions (including the requirement to wear face masks in closed places, proof of vaccination on the Ministry of Health-approved Tawakkalna app is no longer required to enter establishments, events, activities, airplanes and public transport)	13 June 2022

**Table 3:** Hardware and Software Specifications.

	Details
**Processor**	Intel (R) Core (TM) i7-8750H CPU @ 2.20GHz 2.21 GHz
**Storage**	512GB SSD
**Display**	Display1: Intel (R) UHD Graphics 630; Display2:NVIDIA GeForce GTX 1050 Ti
**Software**	Python 3.10.5, Anaconda (Jupyter Notebook 6.4.8, Spyder 5.1.5)
**libraries**	pandas, numpy, matplotlib, statsmodels, sklearn.metrics, fbprophet

**Table 4:** Evaluation of ARIMA used for confirmed cases.

	RMSE	MAE	$R^{2}$
ARIMA (1, 0, 7)	148.228	**74.747**	**0.983**
Baseline	**123.215**	95.674	0.734

**Table 5:** Evaluation of ARIMA used for recovered cases.

	RMSE	MAE	$R^{2}$
ARIMA (1, 1, 0)	239.438	104.609	**0.971**
Baseline	**83.906**	**68.791**	0.861

**Table 6:** Evaluation of ARIMA used for death cases.

	RMSE	MAE	$R^{2}$
ARIMA (0, 0, 9)	1.782	1.544	**0.964**
Baseline	**1.542**	**1.166**	0.913

**Table 7:** Evaluation of the Prophet model used for confirmed cases.

	RMSE	MAE	$R^{2}$
Prophet	175.447	120.728	**0.977**
Baseline	**123.215**	**95.674**	0.734

**Table 8:** Evaluation of Prophet model used for recovered cases.

	RMSE	MAE	$R^{2}$
Prophet	303.542	207.460	**0.931**
Baseline	**83.906**	**68.791**	0.861

**Table 9:** Evaluation of Prophet model used for death cases.

	RMSE	MAE	$R^{2}$
Prophet	1.626	**1.151**	**0.981**
Baseline	**1.542**	1.166	0.913

**Table 10:** Evaluation of all models.

	RMSE	MAE	$R^{2}$
**Confirmed Cases**			
ARIMA (1, 0, 7)	148.228	**74.747**	**0.983**
Prophet	175.447	120.728	0.977
Baseline	**123.215**	95.674	0.734
**Recovered Cases**			
ARIMA (1, 1, 0)	239.438	104.609	**0.971**
Prophet	303.542	207.460	0.931
Baseline	**83.906**	**68.791**	0.861
**Deaths**			
ARIMA (0, 0, 9)	1.782	1.544	0.964
Prophet	1.626	**1.151**	**0.981**
Baseline	**1.542**	1.166	0.913

**Figure A4:**
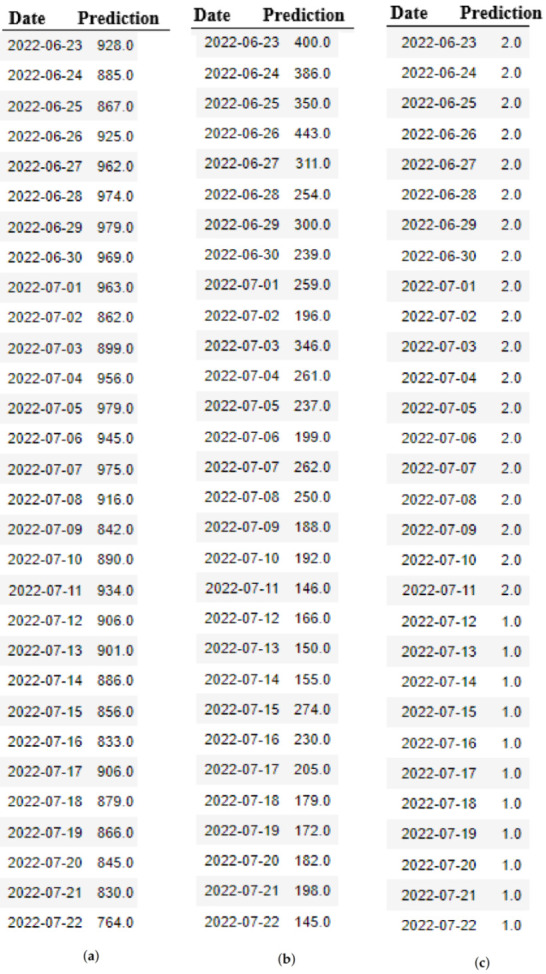
Predicted Future Cases (30 Days) using Prophet. (**a**) Daily cases. (**b**) Recovered cases. (**c**) Deaths cases.

## Data Availability

No new data were created in this study. Data sharing is not applicable to this article.

## Abbreviations

The following abbreviations are used in this manuscript:

ACF	Auto Correlation
ADF	Augmented Dickey-Fuller
AIC	Akaike’s Information Criterion
ANN	Artificial Neural Network
AR	Auto Regression
ARIMA	Autoregressive Integrated Moving Average
FDA	Food and Drug Administration
MA	Moving Average
MAE	Mean Absolute Error
LSTM	Long-Short Term Memory
ODE	Ordinary Differential Equation
PACF	Partial Auto Correlation
PCR	Polymerase Chain Reaction
R${}^{2}$	Coefficient of determination
RMSE	Root Mean Squared Error
RNN	Recurrent Neural Network
RT PCR	Reverse Transcription Polymerase Chain Reaction
SIR	Susceptible Infectious Removed
TS	Time Series

## Appendix A

To see the forecast components, one can use the Prophet−plotcomponents method. By default you’ll see the trend and seasonality. Here, you can see the components (trend, seasonality) of confirmed cases in KSA from 2 March 2020 till 22 June 2022.

To see the forecast components, one can use the Prophet−plotcomponents method. By default, the trend and seasonality can be viewed. Here, you can see the components (trend, seasonality) of recovered cases in KSA from 2 March 2020 till 22 June 2022.

To see the forecast components, one can use the Prophet−plotcomponents method. By default, the trend and seasonality can be viewed. Here, you can see the components (trend, seasonality) of death cases in KSA from 2 March 2020 till 22 June 2022.

## Appendix B

This ﬁgure shows the predicted values of conﬁrmed, recovered and death cases in KSA for next month (from 23 June 2022 till 22 July 2022) using Prophet model.
